# Whole Cell Actinobacteria as Biocatalysts

**DOI:** 10.3389/fmicb.2019.00077

**Published:** 2019-02-18

**Authors:** Yitayal Shiferaw Anteneh, Christopher Milton Mathew Franco

**Affiliations:** ^1^College of Medicine and Public Health, Medical Biotechnology, Flinders University, Bedford Park, SA, Australia; ^2^Department of Medical Microbiology, College of Medicine, Addis Ababa University, Addis Ababa, Ethiopia

**Keywords:** Actinobacteria, biocatalysts, nitriles, biotransformation, biofuel, ethylene glycol

## Abstract

Production of fuels, therapeutic drugs, chemicals, and biomaterials using sustainable biological processes have received renewed attention due to increasing environmental concerns. Despite having high industrial output, most of the current chemical processes are associated with environmentally undesirable by-products which escalate the cost of downstream processing. Compared to chemical processes, whole cell biocatalysts offer several advantages including high selectivity, catalytic efficiency, milder operational conditions and low impact on the environment, making this approach the current choice for synthesis and manufacturing of different industrial products. In this review, we present the application of whole cell actinobacteria for the synthesis of biologically active compounds, biofuel production and conversion of harmful compounds to less toxic by-products. Actinobacteria alone are responsible for the production of nearly half of the documented biologically active metabolites and many enzymes; with the involvement of various species of whole cell actinobacteria such as *Rhodococcus, Streptomyces, Nocardia* and *Corynebacterium* for the production of useful industrial commodities.

## Introduction

Biotransformation is the process by which substrates are converted into useful products using biocatalysts either in the form of whole cells or their enzymes (Ward and Köhler, 2015; Bezborodov and Zagustina, 2016).The classical chemical based transformation of substrates is prone to several disadvantages, including ecologically unfavorable conditions and associated undesirable by-products (Lin and Tao, 2017). Unlike chemical methods, biocatalysts provide several benefits such as their availability from renewable resources, they work at low temperature and pH, are easy to degrade biologically and are selective in both substrate and product stereochemistry (Jemli et al., 2016). Enzyme based biotransformations, however, are also not free of drawbacks including their high cost, higher susceptibility to changes in operating conditions and substrate or product toxicity (Garzón-Posse et al., 2018). In contrast to other catalytic reactions, whole cell biocatalysts allow transformation of substrates via multiple cascades of reactions, help generation of cofactors, have high regio- and stereo-selectivity, work under mild operational, environmentally-friendly conditions, and help selective hydroxylation of non-activated carbon atoms. The latter is not possible with chemical catalysts (de Carvalho, 2017). In addition to these advantages, compounds produced by microorganisms are considered to be safe, which attract many health-conscious consumers (de Carvalho, 2017).

To meet the growing call for efficient and economically feasible biocatalysts, researchers are testing different groups of microorganisms, including actinobacteria (Mukhtar et al., 2017), *Escherichia coli* (Lin et al., 2013; Ladkau et al., 2014; de Carvalho, 2017), *Pseudomonas putida* (Gehring et al., 2016), *Bacillus cereus* (Banerjee and Ghoshal, 2010), *Enterococcus faecalis* and *Saccharomyces cerevisiae* (Whited et al., 2010; Lin and Tao, 2017). Actinobacteria are widely distributed in nature, with several phenotypes including anaerobes, aerobes, spore formers, unicellular, and filamentous forms (Lewin et al., 2016). They are one of the most diverse, well characterized and metabolically versatile group of microorganisms. They play an essential role in maintaining soil structure and carbon recycling through decomposition of various organic matter such as cellulose, chitin, and pectin (Priyadharsini and Dhanasekaran, 2015; Kim et al., 2016). Furthermore, they produce several enzymes (amylases, cellulases, proteases, chitinases, xylanases, and pectinase) (Mukhtar et al., 2017), antibiotics, antitumor agents, plant growth regulators, and vitamins (Prakash et al., 2013; Kamjam et al., 2017).

Over 22,000 biologically active microbial metabolites reported and actinobacteria alone represented 45% of them which are followed by fungi (38%) and unicellular bacteria, especially *Bacillus* sp. and *Pseudomonas* sp. (17%) (Bérdy, 2005; Demain and Sanchez, 2009). Among the described 140 genera of actinobacteria, only few of them produce the majority of active compounds (Jensen et al., 2005; Bull and Stach, 2007; Pimentel-Elardo et al., 2010; Adegboye and Babalola, 2013). *Streptomyces* alone represents three fourth of the total active metabolites produced by actinobacteria (Lam, 2007; Solecka et al., 2012; Barka et al., 2015; Chater, 2016). Table 1 below highlights the approximate share of each microbial group for active metabolite production.

**Table 1:** Microbial share of active bioactive metabolites (Bérdy, 2005).

Source	Total bioactive metabolites	Antibiotics	Other bioactive metabolites
**Bacteria**	**3800**	2900	900
*Eubacteriales*	2750		
*Bacillus* sp.	860		
*Pseudomonas* sp.	795		
*Myxobacter*	410		
*Cyanobacter*	640		
**Actinobacteria**	**10100**	8700	1400
*Streptomyces* sp.	7630		
Other genera	2470		
**Fungi**	**8600**	4900	3700
Microscopic fungi	6450		
Penicillium/Aspergillus	1950		
*Basidiomycetes*	2000		
Yeasts	140		
Slime molds	60		
**Total**	**22500**	**16500**	**6000**


Apart from the above contributions, actinobacteria play a vital part in the development of a sustainable bioenergy industry, predominantly through their cellulolytic enzymes which decompose plant biomass to produce simple sugars that serve as raw materials for biofuel production. Furthermore, their diverse biosynthetic capacity allow them to mediate various environmental interactions which lead to synthesis of various biologically active products (Lewin et al., 2016). Here, we review the application of whole cells actinobacteria for biotransformation of various substrates in a way to produce more active and less toxic compounds as well as biofuels.

## Biotransformation of Harmful Compounds

### Nitrile Biotransformation

Microbial or enzymatic biotransformation of nitriles result the conversion of these toxic compounds into industrially important compounds like acids and amides. Nitriles constitute a group of chemicals widely used in drugs, rubbers, and plastic industries. These compounds contain a cyano group in their backbone which is highly correlated with toxicity (Ramteke et al., 2013). Their high rate of manufacture and continuous usage make them an important source of environmental pollution and have been detected in different environmental samples including sediments of water-treatment plants, in marinas and beach areas (Baxter and Cummings, 2006). Clinically, nitrile toxicity has been associated with cancer and different health problems such as bronchial irritation, respiratory disorders, convulsions, coma, and skeletal deformities (Ramteke et al., 2013). Some researchers highlighted the link with psycho-behavioral abnormalities including learning, memory, motor nerve anomalies in rats treated with aliphatic nitriles such as acetonitrile, acrylonitrile and crotononitrile (Boadas-Vaello et al., 2007; Ramteke et al., 2013).

**FIGURE 1:**
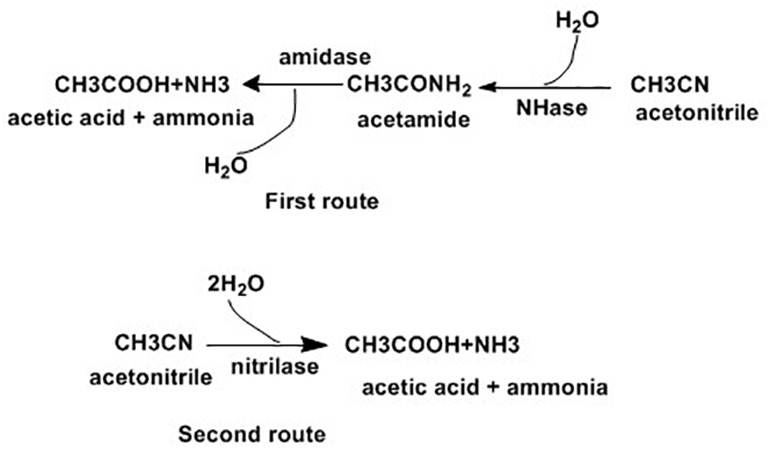
Enzymatic pathways for nitrile hydrolysis (Ramteke et al., 2013).

The removal of nitrile compounds from the environment is possible using microbial methods, due to their associated low cost and user-friendly approach (Fang et al., 2015). Microorganisms degrade nitrile compounds through the hydrolytic route, which comprises two enzymatic systems as indicated in Figure 1. In the first route, nitrile hydratase (NHase, EC 4.2.1.84) catalyzes the formation of amides from nitriles, which are later changed to ammonia and carboxylic acids by amidase (EC 3.5.1.4). Alternatively, nitrilase (EC 3.5.5.1) catalyzes for the direct conversion of nitriles into carboxylic acids and ammonia (Ramteke et al., 2013).

The production of acids and amides from nitriles is possible using chemical catalysts. However, this approach is only achieved under harsh conditions like extreme temperature, acidity or alkalinity (Nigam et al., 2009). Currently, many microorganisms having either of the two nitrile degradation enzymatic systems have been reported. These microorganisms can be categorized into two groups. The first is made up of bacteria like *Mesorhizobium* sp. F28 (Feng and Lee, 2009), *Klebsiella oxytoca* (Kao et al., 2006), *Rhodococcus erythropolis A4* and *Rhodococcus rhodochrous PA-34* (Vesela et al., 2012), which only contain a single enzyme system of NHase/amidase and *Streptomyces* sp. MTCC 7546 only contains nitrilase (Nigam et al., 2009). The other group contain bacteria such as *Nocardia globerula* NHB-2 (Bhalla and Kumar, 2005), *Amycolatopsis* sp. IITR215 (Babu et al., 2010), *Bacillus subtilis* ZJB-063 (Zheng et al., 2008) and *R. rhodochrous* BX2 (Fang et al., 2015), which have both NHase/amidase and nitrilase.

Variation among nitrile degrading microorganisms also exists in terms of the end products of nitrile degradation. Bacteria in the single enzyme system like *R. rhodochrous* PA-34 convert nitrile only into amides while *K. oxytoca* and *Mesorhizobium* sp. F28 convert nitriles into corresponding amides and carboxylic acids. Those bacteria which utilize the two enzyme system result in amides and carboxylic acids. Among these group of bacteria, *R. rhodochrous* BX2, *B. subtilis* AJB-063 and *Paracoccus* sp. SKG (Santoshkumar et al., 2011) displayed completed degradation of carboxylic acids with final end product of ammonia. As indicated above nitrile degradation systems vary among different bacterial genera as well as with in the same genus such as *Rhodococcus.* Unlike others, *Streptomyces* sp. MTCC 7546 in the immobilized as well as Free State biotransforms acrylonitrile into acrylic acid without the formation of amides. The authors suggested that due to several reasons such as operational stability (allow to reuse the system several times), and ease of production on a large scale, the conversion of acrylonitrile using immobilized cells is better than cells in the free state (Nigam et al., 2009).

### Biotransformation of Aromatic Ring Containing Compounds

Phthalate esters and phenols are the two most common chemicals used in industry for stabilization and modification of the characteristics and performance of polymers (He Z. et al., 2014). Di-n-butyl phthalate (DBP), a type of phthalate ester, is a component of different merchandises including pesticides, wrapping materials, makeups, wrappers, wears, and insulators in electric disposals (Dargnat et al., 2009). Similarly, phenol can be applied for the manufacturing of drugs, rubbers, polycarbonate resins, and nylon (Christen et al., 2011).

Phthalates are major environmental pollutants which come into contact with humans and animals through contaminated water systems (He Z. et al., 2014). The European community listed these compounds among the 33 dangerous substances to be controlled in surface water (Dargnat et al., 2009). As they are a constituent of plastics which are now ubiquitous in diverse environments phthalates are now present almost everywhere (Fang et al., 2010). Phthalate toxicity is associated with endocrine system disruption in different species of fish and mammals. These compounds were also observed to interfere with the reproductive system and in human and animal development (Lottrup et al., 2006; Li et al., 2010). Concurrent observation of phenols and phthalate esters has been reported in the Selangor River basin in Malaysia (Santhi and Mustafa, 2013) and induction of lactate dehydrogenase release from Sertoli cells, which is associated with infertility, coexist compared to individual chemical effects (Li et al., 2010).

Different approaches have been documented for removal of DBP from natural environments. These are hydrolysis, photo degradation and biodegradation (Lau et al., 2005; Jonsson et al., 2006; Chen et al., 2009). Two of the former approaches were not effective due to the structural nature of DBP and microbial mediated metabolic transformation of DBP is the current choice. Microbial mediated degradation of DBP involves initial conversion of DBP into phthalic acid and which is further transformed with the help of two dioxygenase pathways into 4,5-dihydroxyphalate and 4,5-dihydroxyphalate in gram negative and gram positive bacteria, respectively. Finally, these two compounds are transformed into a common intermediate protocatechuate under aerobic conditions (Wu et al., 2011). For the degradation of phenols the first step is hydroxylation of phenol to catechol followed by ring cleavage of catechol into 2-hydroxymuconic semialdehyde for the meta-pathway aided by catechol-2, 3-dioxygenase and into cis, cis-muconate with the help of catechol-1, 2-dioxygenase for ortho pathway. Finally 2-hydroxymuconic semialdehyde oxidized 4-oxalocrotonate or hydrolyzed it to 2-oxopent-4-enoate and cis, cis-muconate into muconolactone (Banerjee and Ghoshal, 2010).

Several bacteria strains have the ability to degrade DBP, such as *Rhodococcus* sp. (Yu et al., 2009; Jin et al., 2010), *Gordonia* sp. (Wu et al., 2011), *Agrobacterium* sp. (Wu et al., 2011) and *Enterobacter* sp. (Fang et al., 2010). Members of the *Rhodococcus* genus, have been demonstrated for degradation of Phenol in addition to DBP (Saa et al., 2010), individually as well as via simultaneously mineralization of DBP and phenol (Lu et al., 2009; He et al., 2013). Individual or synchronous biodegradation of DBP and phenol by *Rhodococcus ruber* strain DP-2 was also reported in similar study (He Y.C. et al., 2014).

**FIGURE 2:**
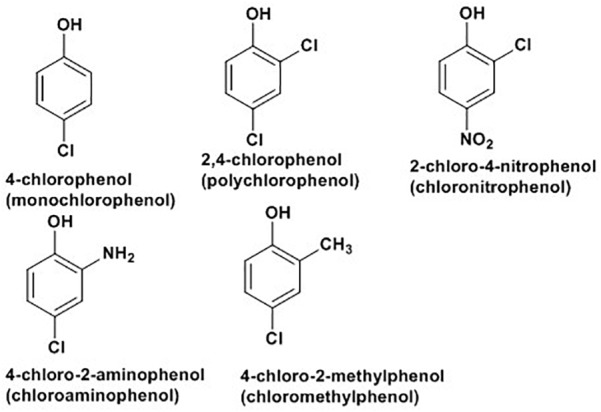
Some examples of chlorophenols (Arora and Bae, 2014).

Chlorophenols (CPs) are aromatic compounds which contain a minimum of one chlorine and a hydroxyl group on the benzene rings. Five types of CPs, as indicated in Figure 2 below, based on chemical structures include monochlorophenols, polychlorophenols, chloronitrophenols, chloroaminophenols, and chloromethylphenols (Arora and Bae, 2014). They are largely used as fungicidal, germicidal and wood preservatives agents. They are also important for synthesis of dyes and drugs (Arora and Bae, 2014). CPs and their derivatives rank among the top environmental pollutants where industrial wastes, pesticides, herbicides, and complex chlorinated hydrocarbons are major sources of contamination (Olaniran and Igbinosa, 2011). Direct skin contact and eating or drinking of contaminated substances are major sources of people exposure (Arora and Bae, 2014). Cellular exposure to CPs are associated with cytotoxic, mutagenic and carcinogenic properties, with several types of polychlorophenols labeled as potential human carcinogens by the World Health Organization and the International Agency for Research on Cancers (Igbinosa et al., 2013).

**FIGURE 3:**
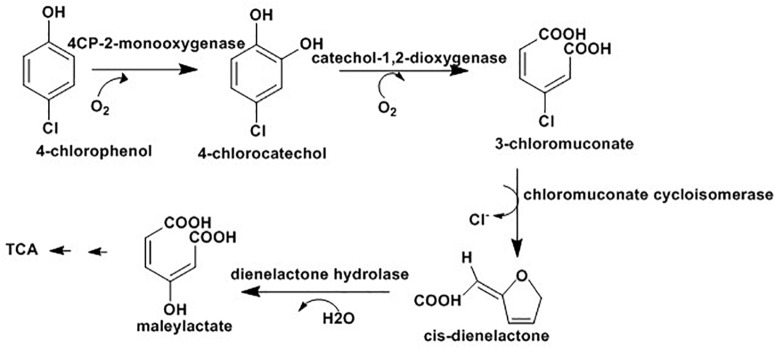
4-chlorophenol degradation via ortho postion where the final intermidate, maleylactate, inter for TCA cycle for complet mineralizatio (Arora and Bae, 2014).

Different possible mechanisms are reported for bacterial degradation of CPs and their derivatives. In the first mechanism, hydroxylation of chlorophenolic rings at ortho-positions with the help of monooxygenases results in formation of chlorocatechols which are further degraded (Hollender et al., 1997; Solyanikova and Golovleva, 2011) or hydroxylated prior to ring cleavage (Nordin et al., 2005). In the second mechanism, with the same enzyme, hydroxylation of chlorophenolic rings at meta position results in chlorocatechols which degrade via hydroxylation (Nordin et al., 2005) or dehalogenation (Xun et al., 1992) prior to ring cleavage. The third mechanism applicable for degradation of chloronitrophenols where the degradation may be initiated by hydroxylation (Arora and Jain, 2012), reductive dehalogenation (Pandey et al., 2011), or reduction of the nitro group (Arora and Jain, 2012). Finally, in case of chloroaminophenols degradation, the pathway may start with removal of ammonium ions by the enzyme deaminase followed by the ring cleavage (Arora and Bae, 2014) or the dehalogenation (Arora and Bae, 2014). The detail mechanisms of different routes of biodegradation of chlrophenols and its derivatives with various bacteria, such as *Pseudomonas knackmussii* B-13, *Rhodococcus opacus* 1G, *Arthrobacter chlorophenolicus* A6, *Streptomyces rochei 303, Pseudomonas* sp. NCIB9340, *Bacillus insolitus, Nocardioides* sp. K44, *Mycobacterium chlorophenolicum* PCP1 and *Mycobacterium fortuitum* CG-2 are well documented in a recent review by Arora and Bae (2014); and the following Figure 3 presents degradation of 4-chlorophenol as an example of the process.

Hou and his colleagues reported for the first time magnetically immobilized *R. rhodochrous* cells for biodegradation of CPs. Their study demonstrated that *R. rhodochrous* DSM6263 depends on constitutively expressed enzymes for hydroxylation of CPs resulting in chlorocatechol formation and complete degradation. Their observation was consistent with another study where *Rhodococcus* sp. AN-22 (an aniline-assimilating bacterium) produced cis, cis-muconic acid from catechol (Matsumura et al., 2006) and highlighted how these compounds could also be metabolized with immobilized cells of *R. rhodochrous* DSM6263 (Hou et al., 2016). Researchers advocated the use of immobilized cells, over free cells, to degrade toxic chemicals due to many reasons such as long-term stability of the catalyst and the immobilization also protects cells from harmful effects of toxic pollutants (Jianlong et al., 2002). Immobilization of cells can be done using a number of techniques including surface adsorption, natural or artificial flocculation, covalent or electrostatic binding to carriers, and encapsulation in a polymer-gel (Hou et al., 2016).

Atrazine (2-chloro-4-(ethylamino)-6-(isopropylamino)-1, 3, 5-triazine) was first introduced in 1950s as an emergent herbicide and they are among widely used pesticides in different countries such as United States, Canada, Africa, and Asia Pacific region (Huang et al., 2003; Jablonowski et al., 2011). Usage of atrazine banned in European countries in 2004 as atrazine concentrations in water surpassed or were estimated to surpass allowable limits (Jablonowski et al., 2011). Due to factors like its widespread utilization as a herbicide and its persistence in the environment, it is common to observe traces of atrazine both in surface and ground water bodies (Gilliom et al., 2006). Traces of atrazine were detected in widely dispersed areas which are far from urban and agricultural areas such as in rainwater in different places (Brun et al., 2008) in fog, arctic ice and seawater.

Contact with atrazine is associated with a serious threat to human and ecosystem health. One of the most notable effects of exposure is endocrine (Solomon et al., 2008; Rohr and McCoy, 2010). Many studies link atrazine with harmful effects on the health of animals and humans, such as sexual abnormalities (demasculinization) in frogs, low testosterone production in rats and higher levels of prostate cancer in workers at an atrazine manufacturing factory (Hecker et al., 2005; Liu and Parales, 2009), and it is also categorized as a group 3 carcinogen according to the International Agency for research on cancer^1^. The above observations indicated that there is cause for concern regarding atrazine residues in soil, groundwater, and surface waters.

**FIGURE 4:**
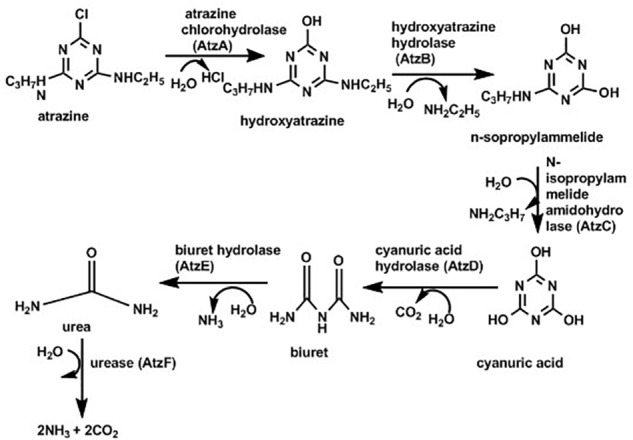
Pathway for atrazine degradation (Liu and Parales, 2009).

Due to its persistence in the environment and being highly toxic, it is very important to develop approaches to degrade and remove atrazine deposits from the environment. Microbial-degradation is one of the methods for elimination of atrazine from soil (Tappe et al., 2002). Different species of microorganisms associated with degradation of atrazine with various degree of biodegradation where some undergo complete mineralization while others produce various intermediates including hydroxyatrazine, deethylatrazine, deisopropylatrazine, n-isopropylammilide, n-ethylammilide, and cyanuric acid (Mandelbaum et al., 1995; Ralebits et al., 2002; Ghosh and Philip, 2004; Getenga et al., 2009). Specifically, atrazine degradation was documented with the help of *Rhodococcus* sp. BCH2 (Kolekar et al., 2014), *Arthrobacter* sp. (Getenga et al., 2009), *Nocardioides* sp. (Topp et al., 2000). *Pseudomonas* sp. strain ADP (Rousseaux et al., 2001; Liu and Parales, 2009) was the first bacterium reported that could completely mineralize atrazine; with most of the degradation studies based on study this strain. As indicated in Figure 4 (below) atrazine degradation is achieved because of the presence of the genes, atzA, atzB and atzC, which code for enzymes such as chlorohydrolase (AtzA), hydroxyatrazine ethylaminohydrolase (AtzB) and N-isopropylammelide isopropylaminohydrolase (AtzC), respectively which convert atrazine sequentially to cyanuric acid (Neumann et al., 2004). Some strains of *Pseudomonas* can further degrade cyanuric acid into CO_2_ and NH_3_.

1, 4-Dioxane is a cyclic ether with many applications including components of deodorants, detergents and various types of paints. The process for manufacturing of polyesters also results 1,4-dioxane production. Various factors including illegal dumping of industrial wastes contribute to 1, 4-dioxane associated water contamination. High level of 1, 4-Dioxane can cause liver and nasal cancers in rats module (Dourson et al., 2014) and are listed as group 2B human carcinogen (Inoue et al., 2016). These compounds are soluble in water with low volatility and have a lower chance of absorbance in solids (Stepien et al., 2014). Therefore, once 1, 4-dioxane appears in the environment it can persist for many days and a high degree of 1, 4-dioxane pollution was observed in surface water, groundwater, and landfill leachatewa (Stepien et al., 2014).

Their removal from water bodies is an important public concern, especially as the routine physical and chemical methods for water decontamination are not effective for removal of 1, 4-dioxane (Adams et al., 1994). Advanced procedures such as the combination of ozone and hydrogen peroxide treatments are expensive to use (Adams et al., 1994; Kishimoto et al., 2008). Thus, cost effective as well as reliable methods for cleaning of 1, 4-dioxane from water lead to nocardioform actinobacteria such as *Pseudonocardia* (Matsui et al., 2016) and *Rhodococcus* (Deeb and Alvarez-Cohen, 1999) which account for the major portion of capable microorganisms. Inoue et al. (2016) tested various species of *Pseudonocardia* and *Rhodococcus* for their ability to degrade 1, 4-dioxane. Their findings indicated *P. dioxanivorans* JCM 13855T (also known as *P. dioxanivorans* CB1190) (Parales et al., 1994) was the only *Pseudonocardia* sp. tested that used 1,4-dioxane as a carbon source and degraded it. In contrast, they observed the inability of *R. ruber* JCM 3205T to degrade 1, 4-dioxane. However, there are reports on the ability of *Rhodococcus* spp. such as *R. ruber* 219 (Bernhardt and Diekmann, 1991) and *R. ruber* T1 and T5 (Oyama et al., 2013) to biodegrade 1,4-dioxane. This highlighted species variation among *Rhodococcus* in relation to effective degradation and utilization of 1, 4-dioxane.

## Production of Important Compounds

### Ethylene Glycol Synthesis

Due to the continuing concern about climate change and depletion of fossil energy, expansion of biological processes using renewable biological resources to produce different chemical feed stocks and energy has become an attractive approach for the chemical industry. Ethylene glycol is a feedstock which serves as a starting material for the manufacture of several items including polymers, anti-freeze agents, and coolants (Zhang and Yu, 2013). Routinely, ethylene glycol is produced through a costly chemical process using ethylene derived from the petrochemical industry as a starting material. Therefore, there is a preference for biological synthesis of ethylene glycol over chemical methods as the former has a low impact on the environment and the reaction is selective (Mattam et al., 2013).

**FIGURE 5:**
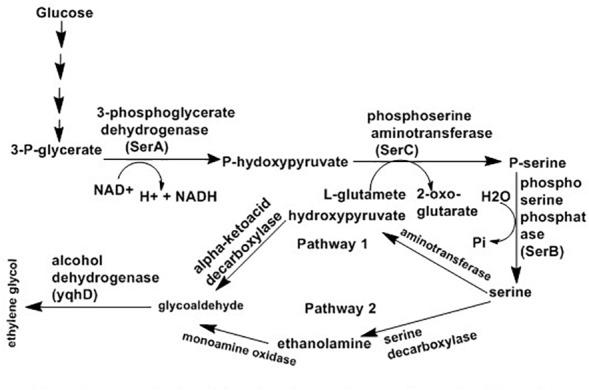
Proposed pathways for synthesis of ethylene glycol from glucose using serine intermediate (Chen et al., 2016).

Fermentation of carbohydrates is an economical process for production of ethylene glycol from biofeed stock. *Corynebacterium glutamicum*, an actinobacterium, has been designed for manufacture of ethylene glycol directly from glucose via extension of the serine synthesis pathway. Serine is produced by most microorganisms from 3-phosphoglycerate (a glycolysis intermediate) using three enzymatic steps: 3-phosphoglycerate converted into P-hydroxypyruvate with the help of 3-phosphoglycerate dehydrogenase (PGDH; *serA*), followed by conversion of P-hydroxypyruvate to P-serine by phosphoserine aminotransferase (PSAT; serC) and P-serine to serine with the help of phosphoserine phosphatase (PSP; serB) (Peters-Wendisch et al., 2005). Chen et al. (2016) proposed two ways for ethylene glycol synthesis via the extension of the serine synthesis pathway where serine is the starting material for both systems with the end product glycoaldehyde (Chen et al., 2016). The first route involves deamination of serine with aminotransferase or amino acid dehydrogenase resulting in hydroxypyruvate. Finally, glycoaldehyde is produce from hydroxypyruvate with the help of α-ketoacid decarboxylase. Similarly, the other route also has two steps where ethanolamide produced from serine with decarboxylation and then converted to glycoaldehyde following oxidation by monoamine oxidase. Finally, glycoaldehyde can be reduce to ethylene glycol by alcohol dehydrogenase such as yqhD as indicated in Figure 5.

In addition to glucose, *C. glutamicum* uses several substrates such as sugars present in molasses (sucrose and fructose), pentose sugars present in lignocellulosic (Zahoor et al., 2012), n-acetyl-D-glucosamine and *n*-acetyl-D-muramic acid (Sgobba et al., 2018) for manufacturing of various amino acids (Bommareddy et al., 2014). Production of other compounds such as isobutanol, cadaverine, and succinate are possible with *C. glutamicum* (Blombach et al., 2011; Buschke et al., 2011; Litsanov et al., 2012).

*Rhodococcus* sp. are also associated with production of Ethylene glycol. *Rhodococcus* sp. CGMCC 4911 converted 1, 3-propanediol cyclic sulfate and its derivatives into corresponding diols. The growing cells of *Rhodococcus* successfully hydrolyzed ethylene sulfate, glycol sulfide, 1, 3-propanediol cyclic sulfate, and 1, 2-propanediol cyclic sulfate with different conversion rates (He et al., 2015).

Cyclic sulfates and its derivatives are very important compounds that serve as starting materials for the synthesis of various useful intermediates (Steinmann et al., 2001). Sulfatases hydrolyse organic sulfate esters into primary or secondary alkyl alcohols (Gadler and Faber, 2007). Microbial transformation of cyclic sulfates into diols can be achieved under mild reaction conditions. He et al. (2013) reported for the first time that *Rhodococcus* sp. CCZU10-1 can convert 1, 3-propanediol cyclic sulfate and its derivatives into diols, where factors like pH, temperature, and cells dose affect rate of biotransformation.

### Production of Less Toxic and Biologically Active Drugs

The absolute configuration of chiral centers in molecules determine the biological activities of the compounds as these molecules bind with receptors made of enantiomerically pure protein (Kato et al., 2003). Most of the available chiral carbon containing drugs are racemic which contain equal concentration of S (+) and R (+) enantiomers. Since only one of the two is active and the other associated with toxicity, the current drug development gives priority to compounds only with a single enantiomer.

**FIGURE 6:**
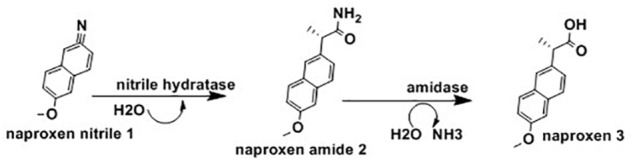
Biotransformation of ibuprofen nitrile 1 to ibuprofen amide 2 and ibuprofen 3 using *Nocardia corallina* B-276 (Lievano et al., 2012).

Ibuprofen [(R,S)-2-(4′-isobutylphenyl)propionic acid] are a group of effective, orally active, nonsteroidal, anti-inflammatory agents which include drugs such as naproxen, fenoprofen, and flurbiprofen (Sen and Anliker, 1996). Even though the S-(+)- ibuprofen form is more than 100 times more effective than the R-(-)-ibuprofen, the racemic form of ibuprofen is widely used (Kumaresan, 2010). The (R)-ibuprofen form can be converted to its enantiomer in the livers and kidneys of pigs and rats, though this process is not free of toxicity (Liu et al., 2009). Figure 6 below highlighted the application of whole cells *N. corallina* to biocatalyse the enantioselective hydrolysis of racemic ibuprofen nitrile [2-(4-isobutylphenyl) propanenitrile, 1] to optically active ibuprofen amide [2-(4-isobutylphenyl) propanamide, 2], a prodrug using nitrile hydratase (NHase) and hydrolyse this amide into optically active ibuprofen (3) using amidase.

The results of the above observation indicated *N. corallina* B-276 displayed nitrile hydratase and amidase activities having low stereo-selectivity (Figure 6). This finding also revealed that *N. corallina* catalyzed deracemisation of racemic ibuprofen to (R)-ibuprofen enantiomer with an efficiency of >99%. This is the first observation where *N. corallina* B-276 catalyzed deracemisation processes. Currently, the (R)-enantiomer form of non-steroidal anti-inflammatory agents are the center of studies and research focus on resolving rac-ibuprofen (Pignatello et al., 2008); and due to this, recent efforts have been directed at resolving rac-ibuprofen (Trung et al., 2006).

A high percentage of optical active S-(+)-ibuprofen was reported from hydrolysis of ibuprofen amide and four related 2-phenylpropionamides using whole cell *Rhodococcus* AJ270 (Snell and Colby, 1999). This high purity was achieved through partial hydrolysis as complete hydrolysis of (R, S)-(+)-2-(4′-isobutylphenyl) propionamide (ibuprofen amide) results in a racemic mixture of ibuprofen enantiomers. The findings of this study suggested that prolonged hydrolysis of ibuprofen amide resulted in both ibuprofen amide and an optical purity of 90-94% of s-(+)-ibuprofen if the reaction is stopped before completion.

Due to its huge production and wide usage, ibuprofen is one of the most commonly detected compounds in wastewater (Buser et al., 1999). In addition to production of active ibuprofen, different species of actinobacteria including *Patulibacter* sp. strain I11 (Almeida et al., 2013) and *Nocardia* sp. NRRL 5646 (Chen and Rosazza, 1994; Cy et al., 2018) are involved in its biodegradation to the level where there is no more risk the community.

Carvedilol is a non-selective, β-adrenergic receptor antagonist and α1-adrenoceptor blocker, and it exists in two enantiomeric forms (Gagyi et al., 2008). The overall cardio-protective action of Carvedilol is due to its (S)-(-)-enantiomer, which is less hepatotoxic than the racemic mixture, and (R)-(+)- enantiomer (Hao and Kim, 2010). Ettireddy et al. (2017) tested *Streptomyces halstedii* and other bacteria for their ability to biotransform racemic carvedilol to its (S)-(-)-enantiomer. The result indicated some bacteria including *S. halstedii* exhibited incubation time dependence enantioselective conversion of carvedilol where the conversion rate increased up to 10 days of incubation and then after the rate is reduced and finally become zero.

**FIGURE 7:**
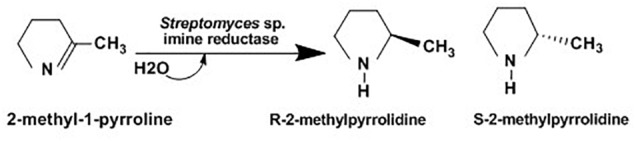
Asymmetric reduction of 2-methyl-1-pyrroline using Streptomyces sp. (Mitsukura et al., 2010).

Chiral amines have been widely used for manufacturing of several therapeutics such as codeine (pain relief), zoloft / sertraline (anti-depression), lariam (anti-malaria) and ethambuto (anti tuberculosis) and agrochemical intermediates including insecticides (imiprothrin, nornicotine), herbicides (imazapyr, imazapic) and fungicides (cyprofuram, fenbuconazole) (Ulrich et al., 2012). Several syntheses of optically active amines have been studied for many years. Asymmetric synthesis of chiral cyclic amine from cyclic imine achieved using whole-cell *Streptomyces sp*. GF3587 and 3546. As showed in Figure 7, these strains produced novel imine reductase enzymes which facilitate enantioselective redaction of cyclic 2-methyl-1-pyrroline into R-2-methylpyrrolidine and S-2-methylpyrrolidine in the presence of glucose.

**FIGURE 8:**
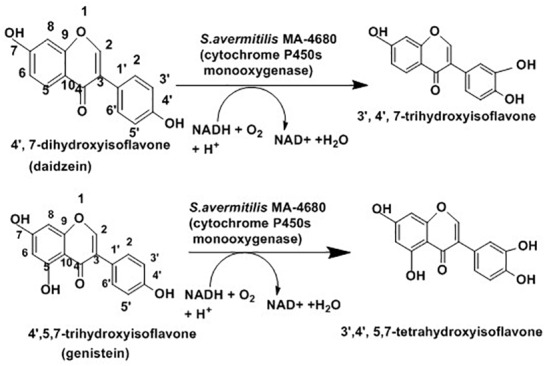
Schematic presentation for isoflavones hydroxylation with the help of *Streptomyces avermitilis* (Roh et al., 2009).

In addition to enantioselective production of active drugs, regioselective addition or substitution of functional groups can also produce active drugs. Actinobacteria play a role in regioselective hydroxylation which mediates the conversion into more active forms. The hydroxylated form of isoflavones such as daidzein (4′, 7-dihydroxyisoflavone) and genistein (4′,5,7-trihydroxyisoflavone) are associated with lowering blood cholesterol and preventing cardiovascular diseases and cancer (Foti et al., 2005). As presented in Figure 8 below, some cytochrome P450 monooxygenases of *Streptomyces avermitilis* MA-4680 catalyze 3′-specific hydroxylation of daidzein and genistein to 3′,4′,7-trihydroxyisoflavone and 3′,4′,5,7-tetrahydroxyisoflavone, respectively (Roh et al., 2009).

**FIGURE 9:**
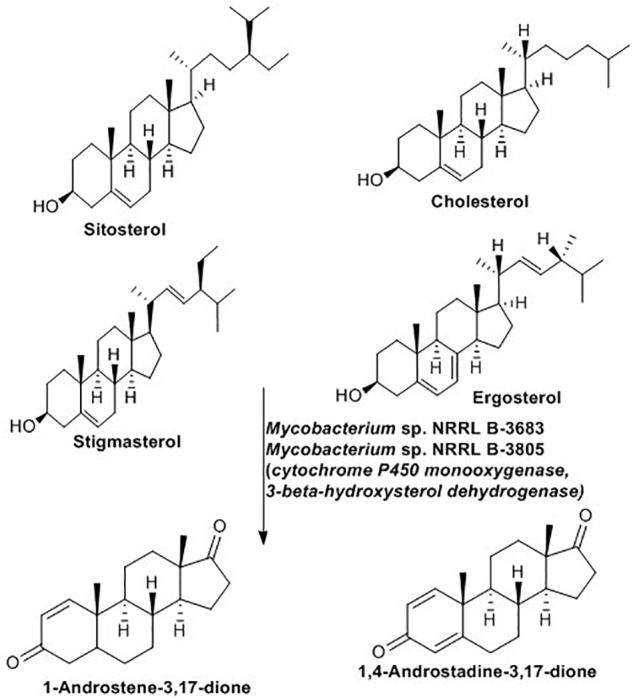
Sterol side-chain cleavage reaction mediated by *Mycobacterium* spp. (Sripalakit et al., 2006).

Microbial biotransformation of natural steroids into pharmaceutically active intermediates has been practiced for many years (Fernandes et al., 2003). These active intermediates play a role in the manufacturing of all type I aromatase inhibitors (Lombardi, 2002) and several high-value steroidal drugs (Wadhwa and Smith, 2000).The bioconversion reaction proceeds with removal of the C-17 side chain of the steroid without any modification of the steroid nucleus (Szentirmai, 1990; Murohisa and Iida, 1993). As indicated in Figure 9, *Mycobacterium* sp. NRRL B-3683 and *Mycobacterium* sp. NRRL B-3805 facilitate a single step C-17 side chain cleavage of sitosterol, cholesterol, stigmasterol and ergosterol to produce C-19 steroids such as 1-androstene-3,17-dione and 1,4-androstadiene-3,17-dione (Sripalakit et al., 2006). The initial step of the side-chain oxidation of sterols is hydroxylation at C-17. The reaction is catalyzed by cytochrome P450 monooxygenase, then several dehydrogenase including 3-β-hydroxysterol dehydrogenase remove hydrogen to introduced a double bound at various points (Donova and Egorova, 2012).

### Amino Acid Production

Amino acids alone or in combination with other molecules can be used as drugs, food supplements, agriculture chemicals, and polymers. Ever year the annual production of amino acids has increased and is currently estimated to be about 3.7 million metric tons (Ikeda and Takeno, 2013). The global market for amino acids is estimated to be about US$6.6 billion and expected to increase by 8-10% each year (Leuchtenberger et al., 2005; Ikeda and Takeno, 2013). Amino acids including L-lysine, DL-methionine, L-threonine, and L-tryptophan account for the largest share in terms of production followed by L-glutamate, L-aspartate, and L-phenylalanine (Ikeda and Takeno, 2013).

**FIGURE 10:**
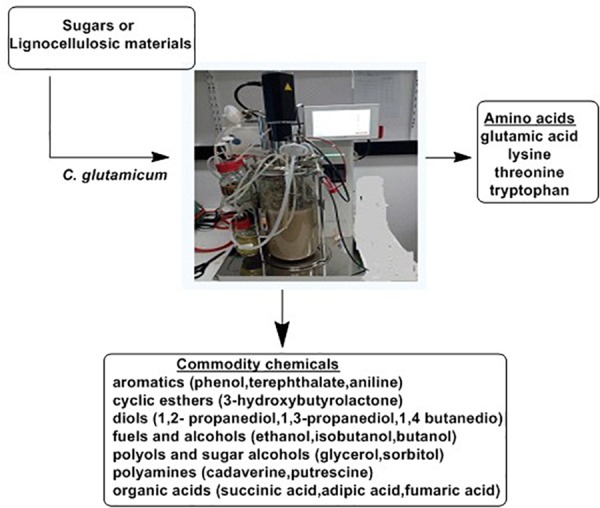
The Biotechnological potential of *Corynebacterium glutamicum* (Ikeda and Takeno, 2013).

Microbial fermentation is the primary source of most of the available amino acids in the market. The L-glutamate-producing bacterium, *C. glutamicum*, has been the cornerstone for the introduction of fermentation in industrial manufacturing of amino acids (Udaka, 1960). This bacterium is still the most widely utilized strain for manufacturing of important amino acids, mainly L-glutamate and L-lysine (Leuchtenberger et al., 2005). Manipulation of metabolic pathways of this bacterium allowed for the an improved product range including L-phenylalanine, L-aspartate, L-tryptophan, L-arginine, L-valine, nucleic acids such as purines, vitamins such as riboflavin and pantothenic acid (Burkovski, 2008; Gopinath et al., 2012) and significant amounts of organic acids such as acetic, lactic and succinic acid (Inui et al., 2004). Figure 10 highlights the biotechnological role of *C. glutamicum*. As indicated, *C. glutamicum* utilizes different starting materials for manufacturing of amino acids and other chemical commodities.

Apart from the above mentioned biologically active compounds, different species of actinobacteria are involved in the synthesis of active compounds. Table 2 below summarizes some of these compounds.

**Table 2:** Industrial production of bioactive molecules using whole cells as biocatalysts.

Compound	Microorganism	Biological activity	Reference
nicotinamide	*Rhodococcus rhodochrous* J1		Meyer and Ruesing, 2008
L-lysine	*Corynebacterium glutamicum*	Amino acids	Burkovski, 2015
L-glutamate	*Corynebacterium glutamicum*	Amino acids	Burkovski, 2015
carboxylic acids	*Rhodococcus* sp. MTB5	Various application	Ismailsab et al., 2017
phenylpropanoic acid	*Nocardia diaphanozonaria* JCM3208	Non-steroidal anti-inflammatory drugs	Mitsukura et al., 2002
aromatic dicarboxylic acids	*Rhodococcus jostii* RHA1	Aromatic chemicals synthesis	Mycroft et al., 2015
ammonium acrylate	*Rhodococcus ruber* NCIMB 40757	Raw materials for water-soluble polymers	Webster et al., 2001
L-malic Acid	*Nocardia* sp.	Metabolites	Hronska et al., 2015
butyramide	*Rhodococcus rhodochrous*	Drugs	Raj et al., 2007
daptomycin	*Streptomyces roseosporus*	Antibiotics	Boeck et al., 1988
hydroxylated adamantine (1-adamantanol)	*Streptomyces griseoplanus*	Pharmaceutical intermediate	Mitsukura et al., 2006


## Production of Biofuels

A recent report indicated 80% of the world’s energy is obtained from fossil fuels such as petroleum, coal, and natural gas (Birol, 2017) with huge consequences for all living systems. Therefore, the search for environmentally benign sustainable energy from renewable sources continues. Plant biomass is considered the most promising feedstock to meet the global demand for sustainable energy and chemicals (Jojima et al., 2013). Lignocellulosic biomass hydrolysates are the primary polymers of plant biomass ideal for biofuel production. Glucose and xylose are the major components of this biomass followed by minor sugars, such as arabinose, and galactose (Elander et al., 2009).

Microbial conversion of plant biomass to sugars and their transformation into a wide array of important compounds, contribute in a major way to the development of a sustainable biofuel industry (Van Hamme et al., 2003; Fulton et al., 2015). Unlike other bacteria phyla, actinobacteria are equipped with the necessary enzymes for degradation of plant biomass (Berlemont and Martiny, 2013) and form the choice of biocatalysts in the biofuel industry (Lewin et al., 2016). Several species of actinobacteria including *Streptomyces, Cellulomonas, Mycobacterium, Propionibacterium, Nocardia, Corynebacterium, Rhodococcus*, and *Micromonospora* are rich in carbohydrate-degrading enzymes (glycoside hydrolase, endo/exo glucanases, cellulases, esterases) (Lombard et al., 2013).

The above mentioned enzymes are used to produce several types of simple sugars, which are further converted into biofuels and other compounds. The widespread utilization of bioethanol from corn and sugarcane and biodiesel from plant oil suffers from several disadvantages (Atsumi et al., 2008; Atabani et al., 2012; Caspeta and Nielsen, 2013). Therefore, alternative biofuels including microbially produced specialty biofuels with similar properties to traditional fuels have increased (Atsumi et al., 2008). These speciality biofuels which encompasses higher-alcohol biofuels, fatty acid alkyl esters and various isoprenoid compounds can be used directly as an energy source or fuel precursors (Peralta-Yahya et al., 2012; Beller et al., 2015). *C. glutamicum* is used for the production of specialty biofuels as this bacterium inherently resists the effect of isobutanol and synthesizes several amino acids like glutamate which are important for production of branched-chain alcohols. Genetic engineering of *C. glutamicum* was employed to produce similar amount of isobutanol as *E. coli* strains, the known producers of isobutanol (Smith et al., 2010; Blombach et al., 2011; Yamamoto et al., 2013). For the past few years, *R. opacus* PD630 has been the center of studies as they were capable of depositing up to 80% lipid in their biomass (Alvarez et al., 2000). The substrates used were alkanes, phenylalkanes, or non-hydrocarbons as the only source of carbon (Hong et al., 2011). Moreover, production of long chain fatty acid alkyl esters (biodiesel) were observed using a *Streptomyces* strain isolated from sheep feces (Lu et al., 2013); bisabolene, another alternative to diesel fuel (Phelan et al., 2014) produced by *Streptomyces venezuelae*; also produced 1-Propanol, an industrially relevant solvent with good fuel properties from *Thermobifida fusca* (Deng and Fong, 2011).

In addition to direct involvement in biofuels production, actinobacteria play a significant role in detoxifying fuel-associated toxic compounds. Starting from formation to maturation, microorganisms have been in contact with crude oil in different reservoirs which contribute for their adaptation to use and modify nearly all chemical categories in crude oil (Mohamed Mel et al., 2015). 85% of fossil fuels contain polycyclic aromatic hydrocarbons (PAHs) and about 13% of these PAHs contain nitrogen, oxygen or sulfur (hetero-PAHs) (Brinkmann et al., 2014). Burning of these fuels results different pollutants such as the oxides of carbon (COx), nitrogen (NOx), and sulfur (SOx) (Ma et al., 2006). Oxides of nitrogen and sulfur combine with water vapor in cloud and result acid rain of sulfuric and nitric acids, which become part of rain and snow (Gupta et al., 2005).

Among PAHs, sulfur (hetero-PAHs) in the form of thiophenic compounds such as benzothiophene (BT), dibenzothiophene (DBT), and their alkylated homologs (Khedkar and Shanker, 2015) have attracted increasingly stringent regulations due to their harmful effects on the environment and human health (Brinkmann et al., 2014; Sousa et al., 2016). The harmful effects of PAHs, particularly hetero-PAHs well documented (Brinkmann et al., 2014) and associated with mutagenic effects, chromosome aberrations, toxicity to daphnia and green algae, embryotoxicity of the zebrafish, and respiratory system irritations.

The concentration of sulfur compounds in crude oil ranges from 0.1 to 8% (w/w) (Müller et al., 2012) and their combustion associated with toxic pollution, most countries developed legislation which demands low level of sulfur in oils, and this in turn forces companies to produce ultra-low sulfur oil, which is currently a challenge (Kawaguchi et al., 2012; Khedkar and Shanker, 2015). Traditionally, reduction of sulfur from crude oil has been achieved with hydrodesulfurization (Gupta et al., 2005), a method that depends on high energy and pressure, which mostly reduces the quality of fuel in terms of energy (Shafi and Hutchings, 2000).

**FIGURE 11:**
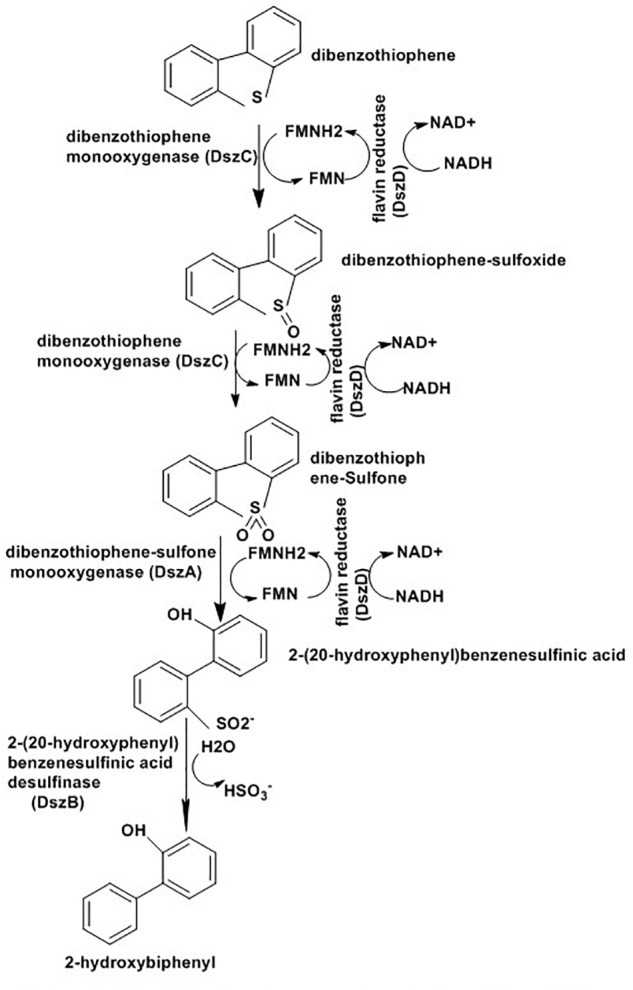
Desulfurization pathway of *R. erythropolis* strain IGTS8 (Gray et al., 2003).

Biodesulfurization depends on whole microbes or their enzymes to eliminate sulfur atom selectively from various refractory compounds present in the fossil fuels. Several strains of *Rhodococcus* sp. (Khairy et al., 2015), *Mycobacterium* sp. (Kawaguchi et al., 2012), Brevibacterium sp. (van Afferden et al., 1990),*Corynebacterium* sp. (Maghsoudi et al., 2001), *Paenibacillus* sp. (Izumi and Ohshiro, 2001), *Pseudomonas* sp. (Van Keulen et al., 1998), *Gordonia* sp. (Chang et al., 2000), and *Bacillus* sp. (Kirimura et al., 2001) have been studied for their ability to metabolize various polyaromatic sulfur heterocycles (PASHs) including BT and DBT (Aggarwal et al., 2013). The process adopted by this organism is known as the 4S pathway and involves four enzymes: DszA, DszB, DszC, and DszD (Wang et al., 2013; Sousa et al., 2016). DszA and DszC are monooxygenases that insert oxygen into the sulfur compounds, while DszB is a desulphinase that removes sulfur in the form of sulfite. DszD supplies FMNH2 to the two monooxygenases and is responsible for reduction of FMN to FMNH_2_ through NADH oxidation to NAD+. The complete reaction as indicated in Figure 11 result in a phenolic product and SO_3_^2-^ (Gray et al., 2003; Kawaguchi et al., 2012).

Most desulfurization studies in the literature have used DBT as the model compound. While several rhodococci strains exhibit non-destructive desulfurization of DBT, *R. erythropolis* IGTS8 (Kilbane and Jackowski, 1992) was the first to be identified and has received the most attention. Even recent study highlighted their potential to desulfurization and denitrogenation of heavy gas oil by *R. erythropolis* ATCC 4277 (Maass et al., 2015). However, most rhodococci are unable to show high activity for the alkyl derivatives of DBT and show no activity for BT and other thiophenic compounds. various *Gordonia* species demonstrated greater desulfurization potential against broader range of PASHs compared to rhodococci (Alves et al., 2005). Of them, *G. alkanivorans* desulfurized DPT with a 4S enzyme system similar to *R. erythropolis.* Besides DBT, it can also specifically cleave the C–S bond in BT and other thiophenes with a reaction rate 2-10 times higher when compared to *R. erythropolis* (Mohebali et al., 2007; Aggarwal et al., 2013).

Microbial mechanisms also operate for degradation of N and O heterocycles. Carbazole is representative N heterocycles and bacteria such as *pseudomonas* sp. and *Rhodococcus* sp. (Maass et al., 2015) reported for mineralization of this compound. Metabolic pathways employed by most microorganisms for carbazole degradation are similar which involve ring cleavage of heterocycles to produce anthranilic acid as intermediates before their complete mineralization. In this pathway, carbazole is first degraded to 2′-aminobiphenyl-2, 3-diol by carbazole 1,9a-dioxygenase which breaks the first C–N bond. This process allow for the selective removal of refractory organonitrogen compounds from petroleum. Unlike other dioxygenases active against aromatic compound carbazole 1,9a-dioxygenase can catalyze cis-dihydroxylation, monooxygenation and angular dioxygenation on diverse aromatic compounds (Xu et al., 2006).

Dibenzofuran is representative of an O heterocyclic pollutant and microbes also developed mechanisms for biodegradation of these O heterocycles. The detail mechanisms were reviewed by Xu et al. (2006) and different monooxygenase and dioxygenases participate in angular dioxygenation, lateral dioxygenation and lateral oxygenation.

## Conclusion

Unlike the traditional chemical based production of biologically active compounds, biocatalysts, particularly whole cell microbial biocatalysts, provide a cost effective and environmentally sound approach. Several candidate microorganisms displayed their ability to catalyze a range of substrates either to change them into usable compounds or to make them less toxic to the general community. Compared to other microorganisms, species in the phylum Actinobacteria are a priority for whole cell biocatalysts as this group of bacteria produce the highest percentage of biologically active compounds compared to any other and they are abundant in a range of environmental conditions. These inherent characteristics of actinobacteria encourage screening of various species in this phylum for their metabolic potential with the discovery of metabolic pathways applicable into different industries, including pharmaceuticals, food and bioenergy sectors. While most screening studies for whole cell actinobacteria as biocatalysts targeted only common species such as *Rhodococcus, Streptomyces* and *Corynebacterium*, future evaluation should consider other uncommon species such as *Gordonia* which has demonstrated promising bio-catalytic activities. Furthermore, most recent studies of whole cell actinobacteria as biocatalysts did not show the detailed mechanisms behind biotransformation such as what genes and enzymes are involved in the process. Future studies should focus on the investigation of the mechanisms behind the biotransformation of certain substrate to product, as this will help to manipulate each system to maximize the yield.

## Author Contributions

CF provided the guidelines and wrote the outline and guided YA in the development of the review, sought a number of research papers on the topic, and did the Tables and the final proofreading. YA did most of the drafting of the manuscript and also sought relevant research papers.

## Conflict of Interest Statement

The authors declare that the research was conducted in the absence of any commercial or financial relationships that could be construed as a potential conflict of interest.

## Footnotes

1 https://monographs.iarc.fr/agents-classified-by-the-iarc/
